# Cytochrome c as a central regulator of mitochondrial function in cancer metabolism

**DOI:** 10.1007/s12672-026-05014-z

**Published:** 2026-04-13

**Authors:** Aisha Shabir, Shazia Sofi, Nusrat Jan, Burhan Ul Haq, Hina Qayoom, Manzoor A. Mir

**Affiliations:** Cancer Biology Laboratory, Bioresources Department, SBS, Kashmir University, Srinagar, India

**Keywords:** Cytochrome c, Metabolic reprogramming, Warburg effect, Electron transport chain, Mitochondrial outer membrane permeability, Apoptosis

## Abstract

Cytochrome c (Cytc) becomes a crucial regulator, determining the fate of cells at the confluence of apoptosis and metabolism. From its primary origin as an electron transporter in the mitochondrial electron transport chain (ETC), Cytc has ascended to a crucial role in apoptosis, triggering cascades of cellular deathupon liberation from the mitochondria.The intricate interplay between Cytc and apoptosis protease-activating factor-1 (Apaf-1) culminates in the apoptosome formation and activation of the cascade of caspase, underscoring the significance of Cytc in regulating cell death pathways. Moreover, tale of Cytc is adorned with post-translational modifications, particularly phosphorylation, which fine-tune its functions in respiration and apoptosis, adding layers of complexity to its regulatory effectiveness. Cytc becomes a lighthouse in the intricate web of cancer, its expression patterns providing hints about prognosis and paths toward treatment. Nevertheless, the story becomes more complex as Cytc becomes entangled in the metabolic reprogramming of cancer cells, implying a crucial involvement in tumor progression and treatment resistance. Collectively, these findings highlight Cytc as a multifunctional regulator of cellular fate that integrates mitochondrial respiration, apoptotic signalling, and metabolic reprogramming, suggesting new opportunities for cancer diagnosis and therapeutic intervention. Even if progress has been made, the story of Cytc is far from over, demanding more investigation into its complexities and biological consequences related to cancer. It represents a therapeutic target in the fight against cancer considering its substantial role in tumor metabolism. It promises a future in which creative solutions to the challenges of cellular destiny will be found. In this review, we have tried to highlight the multidimensional realm of Cytc, connecting threads between apoptosis, metabolic reprogramming, and the Warburg effect in line with cancer.

**Graphical Abstract:**

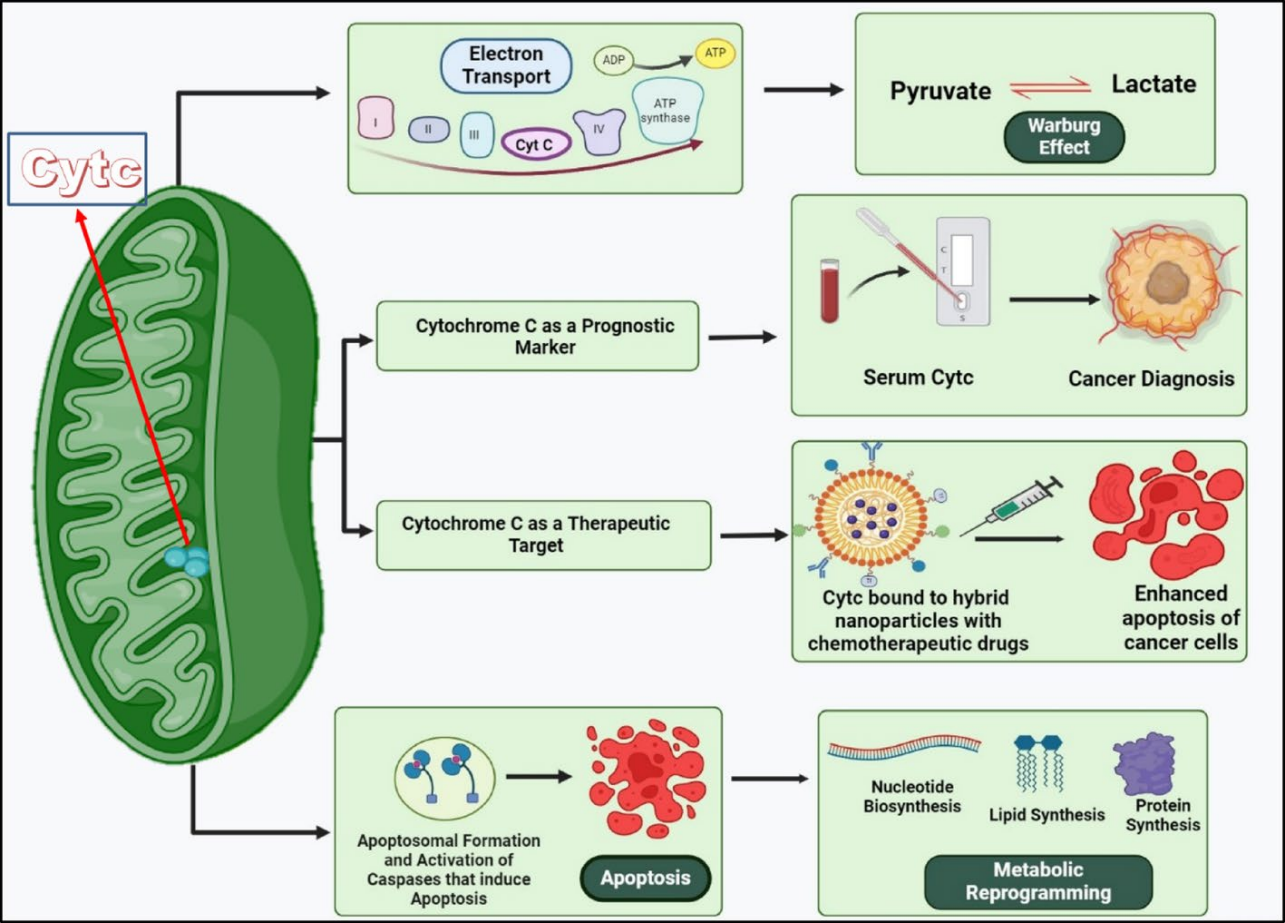

## Introduction

Reliable prognostic markers and treatments with a high degree of sensitivity to cancerous cells are essential for effective cancer therapy. To accomplish these two vital aims, it is necessary to thoroughly characterize the metabolic changes that malignant cells undergo.Chemicals with changed levels, adaptations, or intracellular sites may be uncovered by such changes. One way to measure the effectiveness of cancer therapies is to track how quickly they recover to their pre-treatment levels.Therefore, a therapeutic effect can be achieved by restoring the state of a protein in cancer that has changed in level, alteration, and intracellular position. Cytochrome c has gained recognition as both a valuable prognostic marker and a potential therapeutic target due to its central role in key apoptotic and metabolic pathways. Structurally, it is a small globular protein containing an iron porphyrin cofactor, heme c, covalently bound to a single 104–amino acid polypeptide chain, enabling it to govern critical decisions between cell survival and programmed death. Its fundamental function lies in the electron transport chain on the inner mitochondrial membrane, where it is indispensable for sustaining cellular respiration [1]. It serves as an initiator of apoptosis upon being discharged from the mitochondria [2, 3].Apoptosis, a meticulously regulated process of cellular self-destruction [4], serves as a cornerstone in physiological development, homeostasis, and immune defence, ensuring the removal of unwanted or damaged cells. Central to this intricate machinery is Cytc, initially identified as a crucial mediator of apoptosis through its association with the intrinsic mitochondrial pathway [5].Cytcfunctions as a signaling molecule of apoptosis when liberated out of the mitochondria.Subsequent studies have further elucidated Cytc’s significance, demonstrating its indispensable role in activating downstream caspases and modulating apoptotic cascades [6]. Moreover, Cytc’s release from mitochondria serves as a biomarker for apoptosis [7], offering insights into disease prognosis and treatment response across various cancers. It engages with Apaf-1 and constructs an active apoptosome, activates caspase-9, and initiates the subsequent cascade of caspases [8]. An important inducer of intrinsic apoptosis is Reactive oxygen species (ROS). As a carrier of electrons, Cytc participates in both the elimination and activation of ROS [9].In addition, Cytochrome c catalyzes cardiolipin peroxidation, a process that facilitates its release from mitochondria and promotes apoptosis [2, 3]. Owing to its dual role in oxidative phosphorylation and intrinsic apoptosis, Cytc has become a critical target in cancer cell signaling [10].Within the electron transport chain, it functions as an electron carrier, transferring electrons from complex III to complex IV (cytochrome c oxidase), where oxygen is ultimately reduced to water. This activity is vital for ATP generation and the maintenance of cell survival [11, 12].

Beyond its apoptotic function, Cancer cells show a different metabolic phenotype characterized by increased glycolysis and production of lactate, alongside dysregulated mitochondrial function [13]. Cytc, is situated at the crossroads of these metabolic pathways, it links mitochondrial oxidative phosphorylation with apoptotic signaling pathways and can be indirectly influenced by metabolic reprogramming occurring in cancer cells [14].In contrast to oxidative phosphorylation, most cancer cells predominantly favour aerobic glycolysis, a phenomenon commonly known as the “Warburg effect.” This metabolic shift is part of the cancer cells’ reprogramming strategy to enhance biomass production anabolically, essential for robust cellular proliferation [15].Resistance to cell death and disruption of cellular energetics are prominent features of many cancers and represent key hallmarks of tumor development [16]. Cytc is therefore situated at the centre of pathways governing both of these carcinogenic processes. Recent research demonstrates that Cytc undergoes post-translational modification through phosphorylation in vivo in mammalian heart [17], liver [18], kidney [19, 20], and brain [21] under normal circumstances, occurring on five different residues. These post-translational modifications primarily regulate mitochondrial respiration by modulating electron transfer efficiency and reactive oxygen species production. Although direct effects on apoptosis remain less well characterized, these modifications may indirectly influence apoptotic signalling by altering mitochondrial bioenergetics and redox balance.As of now, there has been noinvestigation that has explored how alterations in the structure and function of Cytc might impact tumor metabolism in cancer at large.Understanding the molecular mechanisms governing functions of Cytc provides novel insights into cancer biology and uncovers probable therapeutic targets for intervention. In the context of cancer, dysregulation of Cytc expression and localization has been implicated in tumorigenesis and disease progression. Studies have revealed altered levels of Cytc in cancerous tissues, suggesting a potential role in modulating apoptotic susceptibility and influencing tumor behaviour. Furthermore, emerging evidence suggests that manipulating Cytc levels or enhancing its release from mitochondria may hold therapeutic promise, offering novel strategies for sensitizing cancer cells to apoptotic stimuli and improving treatment outcomes. Despite significant strides in elucidating role of Cytc in cellular physiology and cancer biology, several questions remain unanswered. Future research endeavours will undoubtedly focus on revealing the complexities of Cytc regulation, exploring its crosstalk with other cellular pathways, and harnessing its therapeutic potential for cancer treatment. By addressing these knowledge gaps, we can elaborate our understanding of Cytc biology and explore possibilitiesfor innovative approaches to cancer therapy.In this review, we aim to comprehensively dissect the multifaceted role of Cytc in apoptosis and metabolism, shedding light on its implications for cancer pathogenesis and therapeutic strategies. Through a synthesis of existing literature and critical analysis, we delineate the intricate molecular networks involving Cytc and propose future directions for research in this burgeoning field.

## Insights into critical involvement of cytochrome c in cellular apoptosis

**Fig. 1:**
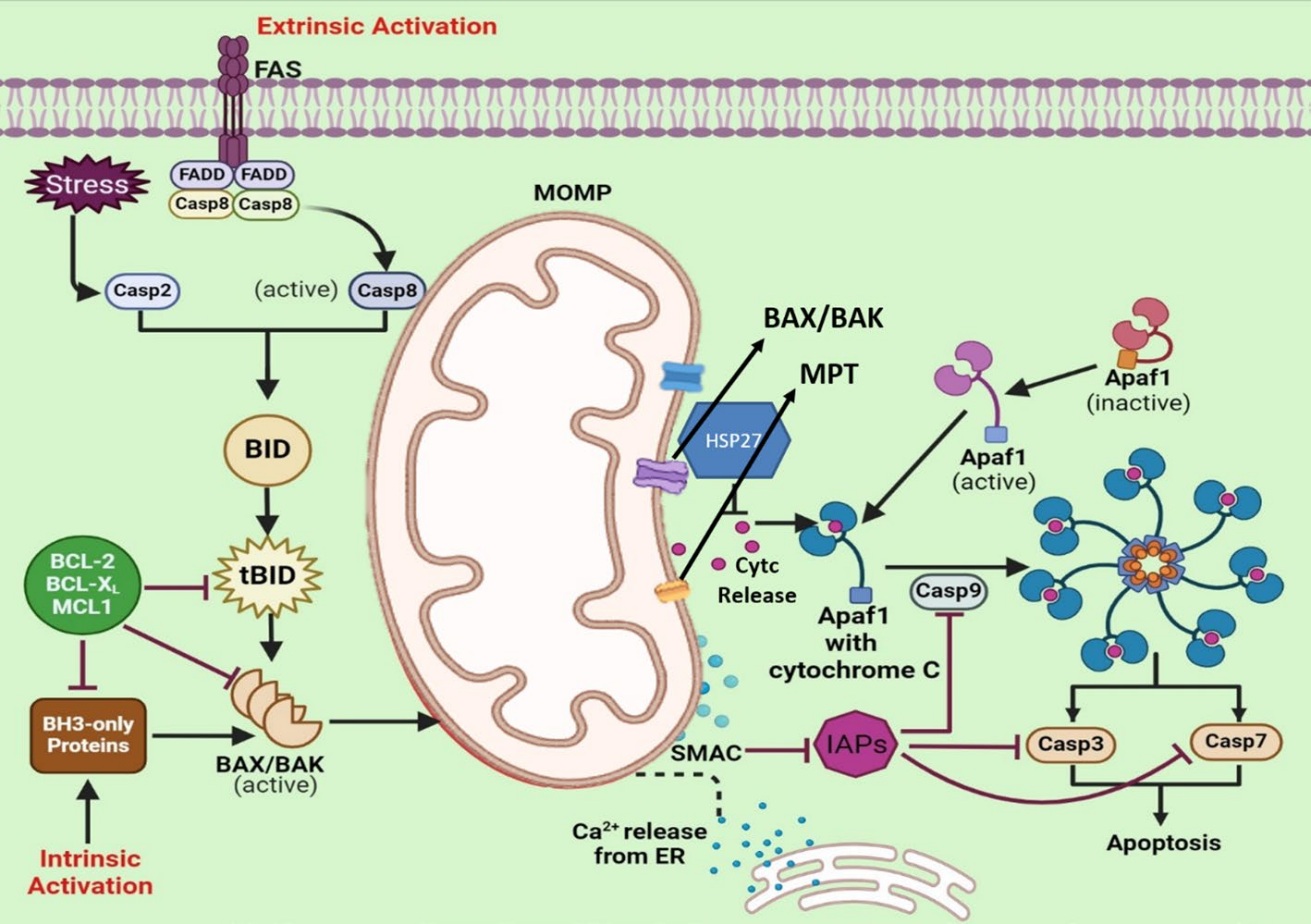
Cytc in Action: Modulating Cellular Processes and Signaling Networks. Under physiological conditions, Cytc and other pro-apoptotic proteins, like the second mitochondria-derived activator of caspase (SMAC), remain confined within the mitochondria. The permeability transition pore (PTP) is closed, preventing the activation of pro-apoptotic BCL2 family members by anti-apoptotic counterparts. Caspases in the cytosol remain inactive, and apoptotic APAF1 is in its auto-inhibited state. Upon exposure to apoptotic stimuli, such as DNA damage or cellular stress, truncated BID (tBID) is generated, activating BAX and BAK to form pores in the OMM. Cytc is then released from the mitochondria through these pores or other channels, entering the cytosol. In the cytosol, Cytc binds to APAF1, facilitating the activation of procaspase-9 and subsequently, caspases-3 and 7. Feedback mechanisms, like the opening of the PTP by releasing Ca2 + from the endoplasmic reticulum (ER), further promote Cytc release. Heat-shock protein-27 (HSP27) inhibits Cytc release, while inhibitors of apoptosis proteins (IAPs) counteract caspase activity. SMAC inhibits IAPs to promote apoptosis. This intricate interplay regulates cellular fate, influencing apoptosis initiation and progression

Apoptosis is one of the major forms of programmed cell death (PCD). It is an essential element of animal existence that eradicates undesired cells, playing a critical role in bolstering immune defence, development of embryo, and maintaining normal body temperatures. The involvement of Cytc in PCD was initially recognized in trials where the caspase activity was prompted by the dATPintroduction to cytosolic extracts [22].The separation of the extracts of cytosol identified Cytc as the constituent of the machinery prompting caspase. The immune depletion of Cytc from an extract eliminated its ability to induce apoptosis, while reintroducing Cytc resulted in the restoration of the potential of PCD.The mitochondrial association was initially noted in an egg-extract system of Xenopus, where unprovoked caspase activation was possible due to mitochondria. Moreover, Cytc was demonstrated to be the primary mediator of this influence [23]. Following that, the cytosolic microinjection of Cytc of diverse cell types of mammals was discovered to initiate apoptosis [24, 25]. Contrarily, cells deficient in Cytc demonstrated resistance to various apoptotic stimuli and displayed reduced activation of caspase-3 [26]. The principal agents of Apoptosis are proteases of cysteine known as caspases, which operate in a synchronized cascade to disassemble the cell**(**Fig. 1**)**. The cascade of caspases encompasses ‘initiator’ caspases followed by ‘executioner’ caspases that can be triggered in distinct manners by various apoptotic triggers [27].

Mitochondrial Cytc functions dually in overseeing both cellular energetic metabolism and apoptosis [28] Mitochondria are widely recognized for their pivotal involvement in energy metabolism, ion balance, and redox control, with sustained harm to these organelles consistently associated with cellular demise [29]. A significant factor contributing to the resilience of carcinomas against radiotherapies as well as chemotherapies is the disturbance of cellular demise pathways like autophagy and apoptosis [30]. This disturbance results in a reduction in the levels of various pro-apoptotic proteins as well as an upsurge of the proteins that inhibit apoptosis. Apoptosis, a form of regulated cellular demise, relies on energy for its execution. In recent times, studies on apoptosis have predominantly concentrated on modifying the mitochondrial respiratory chain instead of modifying the nuclear configuration [31]. Caspase-dependent apoptosis proceeds through two main pathways: the intrinsic and the extrinsic routes [32]**(**Fig. 1**)**.The intrinsic mitochondrial pathway is activated by specific stress signals that induce mitochondrial outer membrane permeabilization (MOMP) and the release of proteins from the intermembrane space. Among these, Cytochrome c plays a pivotal role by initiating caspase activation. Under normal conditions, Cytc resides in the inner mitochondrial membrane, where its heme group enables electron transfer between Complex III and Complex IV in the electron transport chain. However, in response to apoptotic triggers such as DNA damage, metabolic stress, or protein misfolding, Cytc is released into the cytoplasm, where it binds to Apaf-1 to initiate apoptosome formation(Fig. 1**)** [22, 23, 33, 34]. The extrinsic apoptotic pathway is initiated when transmembrane death receptors (such as FAS) bind to their corresponding extracellular ligands (like FASL), resulting in the formation of the death-inducing signaling complex (DISC). This complex subsequently activates initiator caspases, which in turn launch a cascade of enzymatic reactions that drive programmed cell death.

Cytochrome c is central to mitochondria-mediated apoptosis while also functioning as a key component of the respiratory chain that sustains cellular energy metabolism. Liu et al. [22] first identified the role of Cytochrome c in apoptosis. Once released into the cytoplasm, Cytc binds to its adaptor protein Apaf-1 in the presence of ATP, leading to the activation of pro-caspase-9. The activated caspase-9 then stimulates caspase-3, driving the hallmark molecular events of apoptosis through the intrinsic mitochondrial pathway**(**Fig. 1**)** [35].A key early step in apoptosis is the release of Cytochrome c from mitochondria into the cytoplasm. As a core component of the mitochondrial electron transport chain, Cytc is vital for shuttling electrons between Complex III and Complex IV [36].Within an hour of apoptosis triggered by mitochondrial permeabilization, Cytochrome c—an indicator of mitochondrial integrity—is released into the cytoplasm and can also be detected in the extracellular space and bloodstream [37]. As a result, Cytc is recognized as a vital mediator and marker in intrinsic apoptosis**(**Fig. 1**)**. Recent studies indicate that impaired assembly of respiratory chain complexes may sensitize cells to mitochondrial apoptosis by destabilizing electron transport and enhancing ROS production, which promotes mitochondrial outer membrane permeabilization and cytochrome c release.Disruptions in the assembly or regulation of mitochondrial respiratory complexes can profoundly affect cellular bioenergetics and apoptosis. Defects in assembly factors associated with complexes II and IV impair electron transport efficiency, leading to altered mitochondrial membrane potential and increased reactive oxygen species generation. These disturbances may trigger mitochondrial stress responses and influence apoptotic susceptibility, thereby affecting disease progression and clinical outcomes [38].

Current evidence shows that serum Cytochrome c serves as a prognostic indicator in several cancers, including leukemia, lung cancer, and breast cancer [39–42].This indicates that Cytochrome c may play a role not only in the initiation but also in the progression of cancer. A hallmark of malignancy is the evasion of apoptosis, with tumor onset, growth, and metastasis often driven by oncogenic events such as suppression of tumor suppressor genes or activation of oncogenes that interfere with apoptotic pathways [43]. The mitochondrial signaling pathway regulates the release of Cytochrome c and other pro-apoptotic proteins via the Bcl-2/Bax axis, thereby triggering the downstream steps of apoptosis [44–46].Consequently, dysregulation of cytochrome c–mediated apoptotic signaling may contribute to tumor progression by enabling cancer cells to evade apoptosis.

## Cytochrome c liberation: probing cellular relocation

**Fig. 2:**
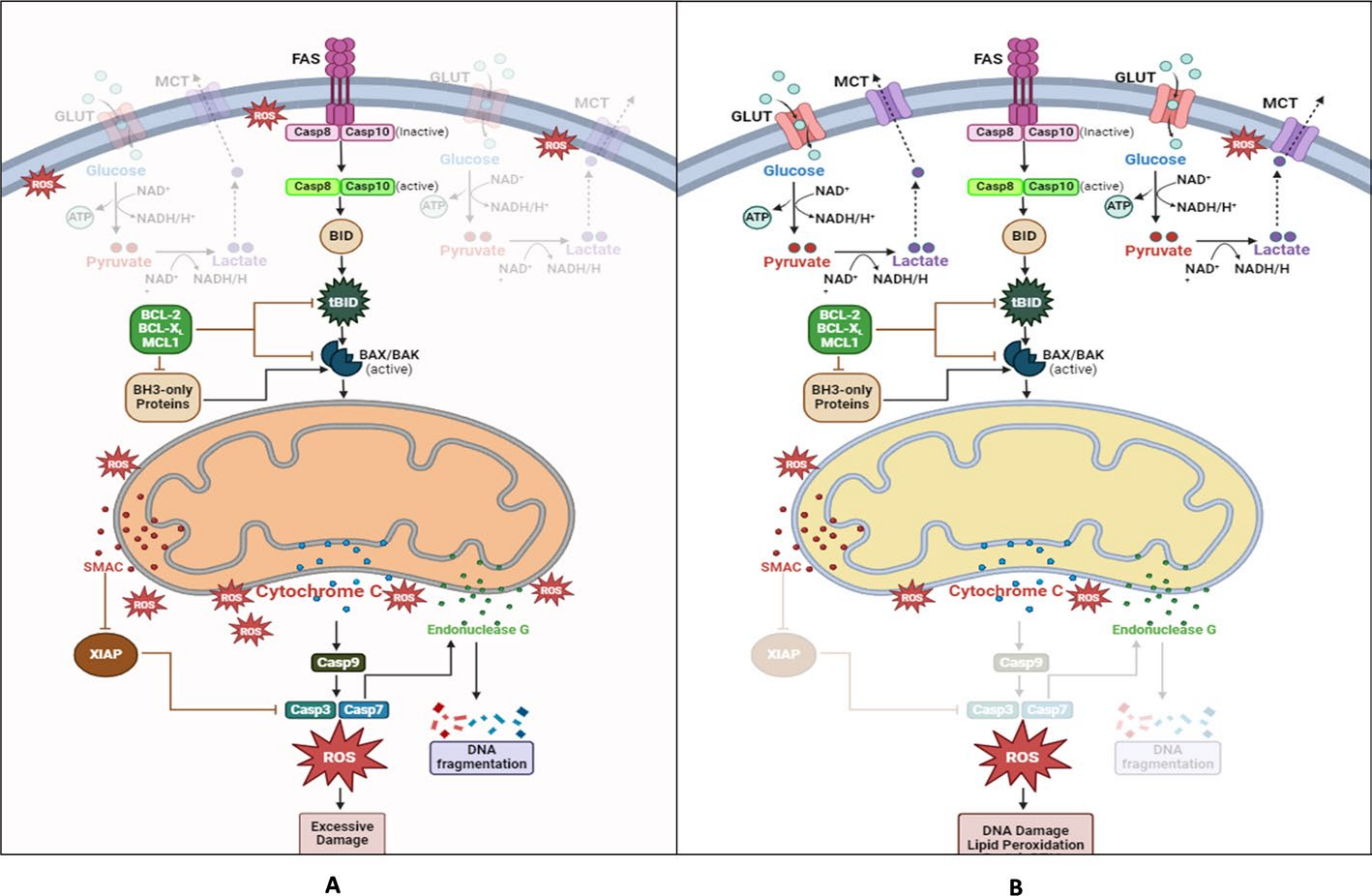
**A **Complete MOMP, triggers the release of SMAC and Cytc, activating executioner caspases and inducing apoptosis efficiently. **B **UnlikeIncomplete Mitochondrial Outer Membrane Permeabilization (iMOMP) which fosters metabolic pathways contributing to drug resistance in cancer, iMOMP leads to a compromised engagement of apoptosis by mitochondria, limiting caspase activity and enabling cells to adapt to mild mitochondrial damage. While SMAC and Cytc are released during iMOMP, executioner caspases remain inactive. This partial permeabilization results in limited membrane depolarization, leading to suboptimal ETC activity and the generation of sub-lethal ROS, which can promote tumor growth. Glycolysis persists and compensates for the reduction in ETC function, thus enabling cancer cells to evade cell death and metabolic crisis, ultimately fostering resistance to therapy and treatment failure

In the course of mobilization, Cytc disconnects from the IMM and disengages from cardiolipin, a membrane phospholipid, through oxidation of cardiolipin [47, 48]. Caspase-2 disturbs the connection between cytochrome c and cardiolipin within the IMM [48], possibly amplifying the mobilization of cytochrome. The movement of Cytc can be intensified by the additional generation of ROS [12, 49]. In case of deficiency of Cytc within the vicinity of mitochondria results in respiratory dysfunction [50]. which may trigger an instant surge in ROS production [51].The mobilization of Cytc could also entail its departure from tight junctions of cristae **(**Fig. 2**)**. To explain the swift and wide-rangingdischarge of Cytc during apoptosis [52], it has been proposed that cristae undergo remodeling, thereby redistributing cytochrome c within the mitochondria before its translocation through the OMM [53].The subsequent stage of Cytc release involves transferring the mobilized protein from the OMM to the cytosol **(**Fig. 2**)**.Death agonists from the BCL2 family induce MOMP and thus manage mitochondrial integrityand consequently oversee the release of Cytc and intrinsic mitochondrial apoptosis [54]. The effector proteins of the BCL2 family such as BAX and BAK are indispensable and hence adequate for MOMP, and MOMP doesn’t transpire in their absence [55, 56]. Heat shock protein-27 (HSP27) inhibits the intracellular movement of BID, thereby impeding the release of Cytc, possibly by protecting the filamentous actin network [57]. **(**Fig. 2**)**. HSP27 attaches to Cytc in the cytoplasm, thereby halting its subsequent impacts. Additional heat-shock proteins, like HSP70 [12] and HSP72 [58] have similarly been demonstrated to affect the discharge of Cytc.Calcium ions (Ca2+) are discharged from the ER, initiating the activation of the Permeability transition pore and a broader release of Cytc [50, 55]. In numerous cell varieties, the liberation of Cytc during PCD occurs swiftly, fully, and irreversibly [52].

The liberation of Cytc during MOMP is believed to be biphasic**(**Fig. 2**)**. Initially, there is the discharge of a ‘loosely tethered’ reservoir, followed by an amplification loop and the remodeling of mitochondrial cristae, eventually prompting the release of the more firmly tethered reservoir [12, 53].The ‘soluble’ Cytc pool, loosely tethered, is accountable for electron transport and the management of ROS. In other words, it plays a significant role in mitochondrial OXPHOSand the generation of ATP. Intriguingly, these processes aren’t notably disrupted until the release of the second wave of Cytc, indicating the potential for metabolic compensation during incomplete MOMP**(**Fig. 2**)**.The ‘firmly tethered’ pool also engages with CL, aiding in the connection of Cytc with mitochondrial membranes. The enhancement following the initial release can stimulate CL oxidation, leading to subsequent detachment from Cytc, activation of caspases, and the consequent cleavage of vital components within ETC complexes. This, in turn, intensifies additional ROS production and results in respiratory collapse [12]. As per the findings, after the liberation of Cytc, caspase-3 initiates cleavage of the p75 subunit (NDUFS1) within Complex I. This leads to an additional decline in potential of mitochondrial membrane and integrity, heightened production of ROS, impairment of the plasma membrane, and a fatal decrease in levels of ATP [59].Hence, the extent of Cytc release not only governs the levels of oxidative metabolism and ATP production but also influences apoptotic involvement. This highlights that the levels of MOMP play a crucial role in adjusting the equilibrium between metabolic as well as apoptotic responses to cellular stress. Figure 2depicts theprocedures and outcomes of both partial MOMP and complete.

## Cytochrome c and metabolic alterations: examining the connections to Warburg’s principle

The metabolic reprogramming in tumor cells is increasingly acknowledged as a significant catalyst for disease, governing multiple facets of malignant advancement [60]. Genetic mutations associated with metabolism of mitochondrial, trigger the stress response pathway, thus contributing in the initiation of tumorigenesis [61]. Elevated generation of mitochondrial ROS as well as alterations in the redox state of cellcan modify the function of transcription factors that promote the development of cancer cells [62]. Defects in mitochondrial function and dysregulated expression of metabolic regulators have been reported across multiple cancers, including gliomas [63], prostate [64], and breast cancer [42].Reprogramming of energy metabolism in tumor cells is considered a key driver that sustains cancer cell proliferation at metastatic sites [42]. Molecules of the mitochondrial electron transport chain (ETC) play a central role in tumor cell metabolism. The ETC drives oxidative phosphorylation, producing energy for ATP synthase, but also serves as a site where electron leakage can generate superoxide, contributing to oxidative stress. The terminal complex, cytochrome c oxidase (COX), transfers electrons from Cytc to oxygen and acts as a key regulatory point of OXPHOS. In many rapidly proliferating tumors, altered mitochondrial function leads to increased glucose consumption as a major energy source [65, 66]. Different cancer types display varied bioenergetic adaptations, with some favoring enhanced glycolysis while others rely more on channeling substrates through oxidative phosphorylation (OXPHOS) [67]. Consequently, the scrutiny of tissue-specific mitochondrial traits is crucial for comprehending the metabolic shifts taking place in cancer cells.

**Fig. 3:**
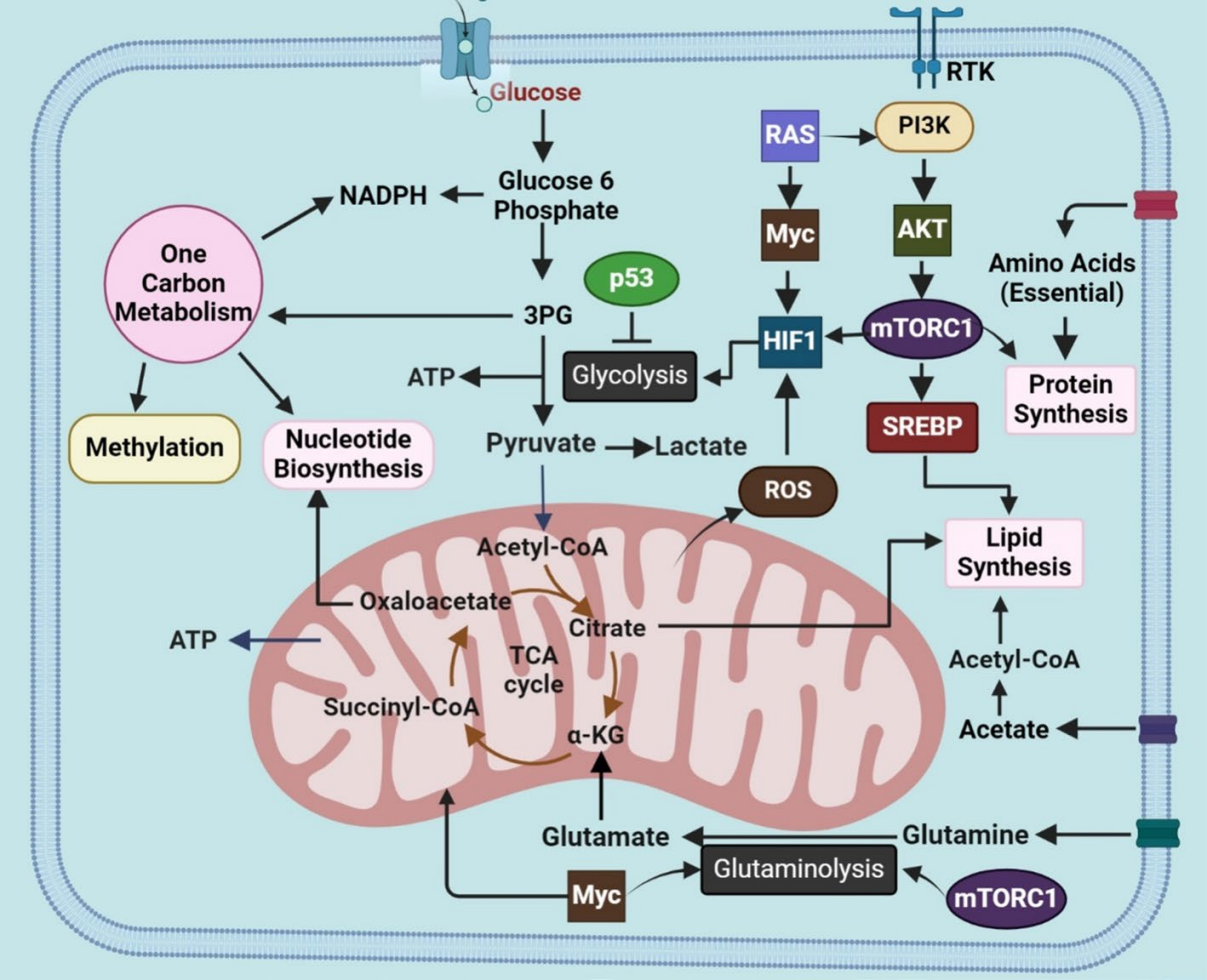
The metabolic reprogramming in cancerous cells is depicted. The main features of the metabolic alterations in cancer cells are portrayed in the picture, including glutamine metabolism, glycolysis, lactate fermentation, the TCA cycle, the pentose pyruvate route, and the synthesis of lipids, amino acids, and nucleotides using TCA cycle intermediates

Glucose metabolism is the most prominently altered metabolic pathway in cancer cells [68]. Since then, many additional pathways have been recognized as altered in cancer, including fatty acid oxidation, lipid uptake, glutamine metabolism, one-carbon metabolism, branched-chain amino acid metabolism, and the Krebs cycle [69]**(**Fig. 3**)**.The reprogramming of these pathways involves intricate mechanisms and the coordinated action of various signaling molecules, including those previously considered insignificant, such as non-coding RNAs. Hypoxia within the tumor microenvironment (TME) drives metabolic changes in cancer cells, including the Warburg effect. These hypoxia-induced alterations in the TME trigger transcriptional responses via HIF activation, ultimately reshaping metabolic pathways in cancer cells [70]. At the cell membrane, PI3K phosphorylates and activates AKT (Fig. 3), a key signaling molecule that drives the Warburg effect in cancer cells [71]. AKT-mediated activation of mTOR promotes lipid synthesis and facilitates glucose transport into cancer cells, thereby boosting glycolysis. By enhancing glucose uptake, glycolysis, and lactate production, the PI3K/AKT pathway plays a central role in the metabolic reprogramming of cancer cells [72]. Glutamine acts as an alternative carbon source for biosynthetic pathways, supporting fatty acid synthesis. Glutamine-derived α-ketoglutarate (Fig. 3) drives citrate production through the forward flux of the TCA cycle and contributes to pyruvate formation via malic enzyme-dependent pathways [73].Additionally, due to the intricacy of these mechanisms, the pathways of metabolic reprogramming in cancer often transpire into diverse extents and contexts across various carcinomas, granting cancer cells flexibility not seen in normal cells [74]. While normal cells usually experience PCD as part of the normal cell cycle, cancerous cells elude the process of apoptosis to augment their expansion, development, and maturation, especially in conditions of low oxygen. A multitude of pathways employed by cancer cells to evade apoptosis are intricately tied to metabolic reprogramming. The metabolic reprogramming observed in cancer, characterized by heightened glycolysis, particularly in glucose metabolism, stands out as a principal feature [75]. The metabolismof glucose is intricately connected to the avoidance of apoptosis through various pathways. Several identical signaling molecules engaged in the elevation of glycolysis also play a role in the inhibition of apoptosis.Cytc is one of the molecules engaged in glucose metabolism and plays a role in suppressing apoptosis.Cytc initiates the mitochondrial apoptotic pathway but undergoes alterations due to the metabolism of glucose. The heightened flow in the pentose phosphate pathway (PPP) in cancer cells leads to an elevated generation of NADPH, inhibiting the activity of Cytc by maintaining it in its inactive reduced state [76].Glycolysis in cancer cells is additionally controlled by Akt, which impedes PCD by restraining two BCL-2 pro-apoptotic family proteins, namely glycogen synthase kinase 3 (GSK-3), p53 upregulated modulator of apoptosis (PUMA).Other molecules participating in the metabolism of glucose and contributing to the inhibition of apoptosis comprise tp53-induced glycolysis and apoptosis regulator (TIGAR) as well as BCL2-associated agonist of cell death (BAD) [77].

Two traditional characteristics of cancerous cells include a metabolic shift from OXPHOS to glycolysis, famously referred to as the Warburg phenomenon, and resilience against cellular demise, and Cytc is situated at the confluence of both pathways. The Warburg Effect posits that cancerous cells mainly utilize aerobic glycolysis, where glucose is converted into lactate in the presence of oxygen, for the production of ATP [78]. This is in contrast to normal cells, which primarily depend on OXPHOS for the production of ATP [79]. Since the proposal of Warburg’s hypothesis, there has been a substantial development in understanding the role that aerobic glycolysis plays in cancerous cells. Specifically, it has been observed that there exists an increased ratio between oxygen utilization as well as glycolysis, a phenomenon orchestrated by changes in oxidative metabolism, the suppression of genes that suppress tumors, and the activation of such genes that promote cancer [80]. Metabolism of Glucose is deemed a crucial facet of cancerous cells, providing building blocks for various essential metabolic pathways, such as the synthesis of nucleotides, lipids, and amino acids [15].

**Fig. 4:**
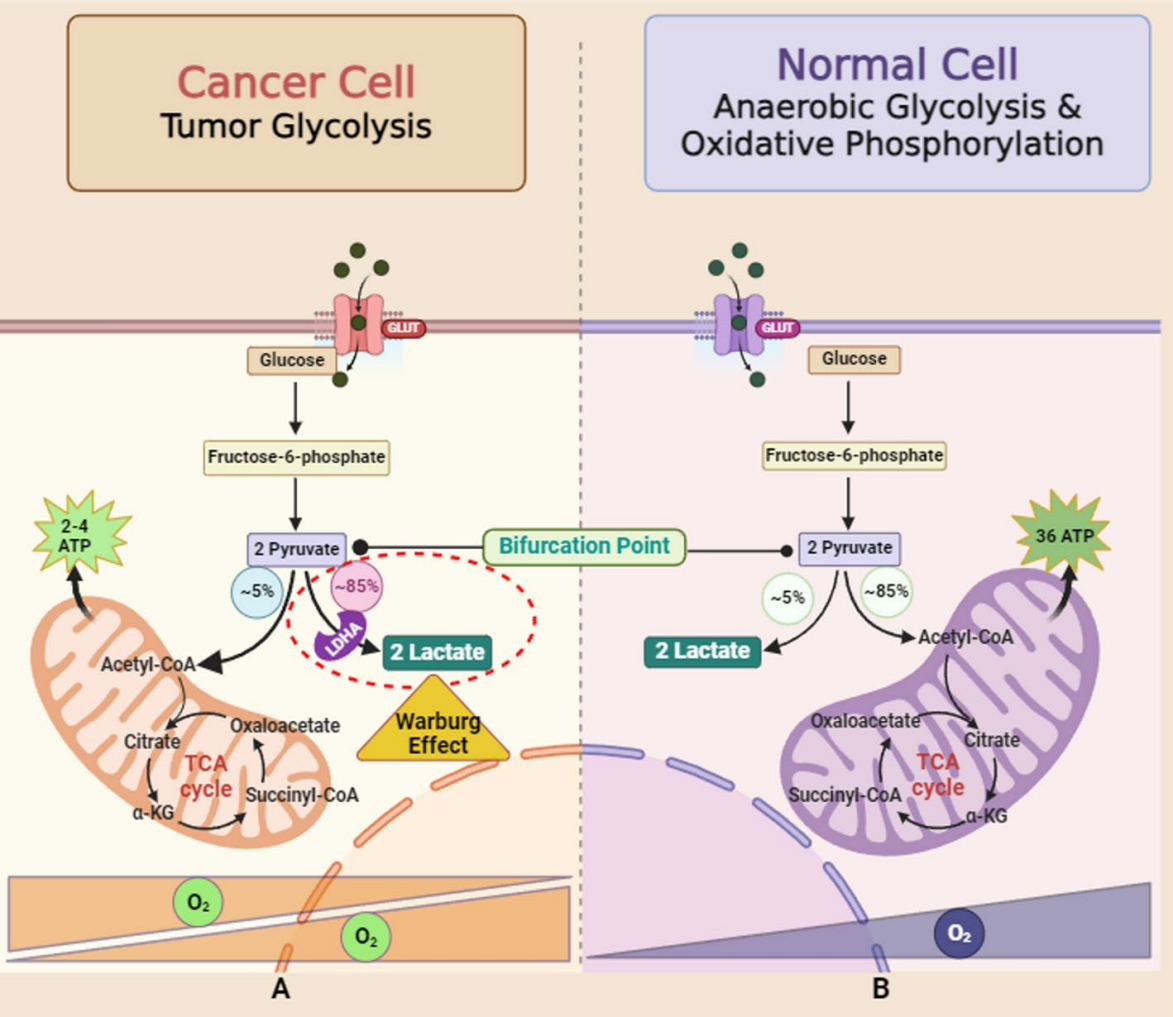
**A **Even in the presence of oxygen, tumors and other highly proliferative cells prefer to convert most of their glucose to lactate, which yields around 4 mol ATP/mol glucose. This process, known as the Warburg Effect, gives cancer cells a significant growth advantage over OXPHOS since it is a faster chemical reaction even though it produces less ATP/mol glucose. **B **One of two paths is used in normally differentiated tissues. Glucose is converted to pyruvate when oxygen is present, and pyruvate then enters OXPHOS to make about 36 mol ATP/mol glucose. Lactate is produced when glucose is not oxidized, producing two milligrams of ATP for every milligram of glucose

Thus, a key question in cancer cell glucose metabolism is why these cells prefer a relatively inefficient energy-generating pathway: while oxidative phosphorylation (OXPHOS) produces 36 ATP per glucose (Fig. 4B), glycolysis generates only 2 ATP (Fig. 4A). The answer lies in the fact that the rate of cytosolic synthesis of ATP is approximately 100 times faster (ranging from 20 to 300 times) compared to mitochondria, which is characterized by “high-speed ATP production, but low yield [81]. Therefore when cancer cells require a significant amount of ATP, aerobic glycolysis can quickly rise, whereas OXPHOS remains relatively stable. This is because the Warburg effect allows for considerably faster ATP synthesis (although glycolysis yields less ATP per glucose molecule than oxidative phosphorylation, it can generate ATP at a faster rate, which may support rapid proliferation of cancer cells) [69]. Rapid energy production is essential for cancer cells to sustain their uncontrolled growth, and aerobic glycolysis efficiently meets this demand. Additionally, the high lactate output acidifies the microenvironment, favoring cells that tolerate acidity. This provides a growth advantage, enhancing the invasiveness and proliferation of cancer cells while surrounding cells deteriorate [75].Extracellular acidosis, defined by a pH below 6.8, arises from both aerobic and anaerobic glycolysis. This acidic environment promotes cancer progression and contributes to resistance against conventional therapies [82].Cancer cells require significant quantities of glucose to maintain energy homeostasis.

The Warburg effect is characterized by many key functional as well as molecular processes such as a significant elevation in the rate of glycolytic flows, the production of sufficient ATP within a given time period to sustain the equilibrium of energy, the redirection of intermediates from glycolytic processes to support the production of lipids, hexosamines, non-essential amino acids as well as nucleotides [83].It also involves the blocking of pyruvate from entering mitochondria and an elevated level and build-up of lactate. Lactate not only promotes the growth of tumor and suppresses anti-tumor immunity, but it may additionally function as a source of energy for cancer cells in oxygen-rich environments thereby leading to malignancy and obstruction to traditional treatments [84]. A metabolic program of cancer cells drives prolonged growth and accelerates aggressive progression; this is reflected in the Warburg effect [81].The Warburg effect facilitates the provision of reducing equivalents, which in turn promotes the development of cancer cells. The oxidative branch of the PPP generates 2 NADPH molecules for each glucose molecule. This helps to maintain the antioxidative capacity of glutathione, which in turn increases the resistance of cancer cells to radiation. Additionally, NADPH may also function as an antioxidant in the mitochondria [85]. In addition, the activated PPP can reduce the generation of ROS, which, at lower levels, can enhance the viability of cancer cells. Furthermore, NADPH is utilized in the process of reductive biosynthesis during the creation of fatty acids, which are essential for the production of membrane lipids [86].

The conversion of glucose to lactate despite the presence of oxygen and functional mitochondria, a phenomenon known as the Warburg effect [87],is undoubtedly more than just an adaptation to hypoxia. On the contrary, it is an urgent component of cancer’s phenotype and a key aspect of cancer cells’ “selfish” metabolic reprogramming, a trait sometimes referred to as a “hallmark of cancer.“ [16]. Hence the alteration of glucose metabolism in cancer therefore not only boosts cancer progression through pathways that stimulate growth but also through mechanisms that prevent cell death and fortify survival.

Mitochondrial dynamics, including the balance between fission and fusion, play a crucial role in regulating cancer cell metabolism. increased mitochondrial fission, mediated by proteins such as dynamin-related protein 1 (DRP1), has been associated with enhanced glycolytic metabolism, mitochondrial fragmentation, and adaptation to hypoxic tumor microenvironments. Conversely, mitochondrial fusion can promote oxidative phosphorylation and improve mitochondrial respiratory efficiency.These structural changes influence mitochondrial function, redox signaling, and the susceptibility of mitochondria to release cytochrome c during apoptosis [88, 89].

The study by Ghosh et al. [90] demonstrated that disruptions in mitochondrial dynamics within the tumor microenvironment significantly affect cellular metabolic programs and immune cell function. Alterations in mitochondrial fission and fusion processes modify mitochondrial bioenergetics, redox balance, and metabolic flexibility, enabling tumor cells and surrounding immune cells to adapt to hypoxia and nutrient fluctuations within the tumor microenvironment. These dynamic changes influence energy production pathways and contribute to metabolic reprogramming observed in cancer progression.

Similarly, findings reported in Biochemical and Biophysical Research Communications [91] emphasize that mitochondrial structural remodeling can regulate metabolic signaling pathways and determine whether cells rely on oxidative phosphorylation or glycolysis. Changes in mitochondrial morphology affect mitochondrial membrane potential, reactive oxygen species generation, and substrate utilization, thereby promoting the metabolic switch toward aerobic glycolysis under oncogenic conditions.

Collectively, these findings suggest that mitochondrial dynamics are not merely structural adaptations but are active regulators of cancer metabolism. Increased mitochondrial fragmentation and altered mitochondrial quality control mechanisms facilitate metabolic flexibility and support the glycolytic phenotype characteristic of rapidly proliferating tumor cells.

## Post-translational modifications of cytochrome c

Alterations in mitochondrial function have been observed in several diseases, including cancer, where changes in mitochondrial metabolism and signalling contribute to tumor progression [92].A pivotal protein in the functioning of mitochondria and regulation of oxidative signaling is Cytc [93].The heme group and four α-helices of Cytc, a tiny soluble protein of 104 amino acids and a molecular weight of around 12 kDa, are covalently bonded by two cysteine residues (Cys14 and Cys17). The heme group is hardly exposed to the solvent since it is encased in a hydrophobic fissure. Cytc can effectively exchange electrons with its redox partners all credits to its shape. Several Cytc residues experience post-translational changes that provide a more precise functional control [2]. Cytc is a component of redox metabolism and is found in the intermembrane gap of the mitochondria in the absence of stimulus. Nonetheless, several triggers—like signals from DNA damage or apoptosis—incentivize the release of Cytc from mitochondria. APAF-1 was the first extra-mitochondrial target for Cytc that was identified [94]. One of the first steps in the mitochondrial apoptotic process is the cytosolic interaction between Cytc and Apaf-1. A wide range of proteins found in many organelles and the cytoplasm are included in the mitochondrial and extra-mitochondrial network of Cytc [95] Such interactions have the potential to influence the choice amongst a cell’s life and death [96], which has implications for well-being as well as illness. Protein modifications following translation serve as regulatory systems that govern a wide array of cellular metabolic events and facilitate the functional diversification of proteins [97]. PTMs are indispensable for the regulation of Cytc functions. As a result of undergoing various PTMs, physicochemical characteristics, connections to physiological associates, and functions of the heme protein are altered. Nevertheless, the specific residues that undergo PTMs influence these outcomes [3, 98].

## Differential cytochrome c expression in various tumor types: insights and patterns

**Fig. 5:**
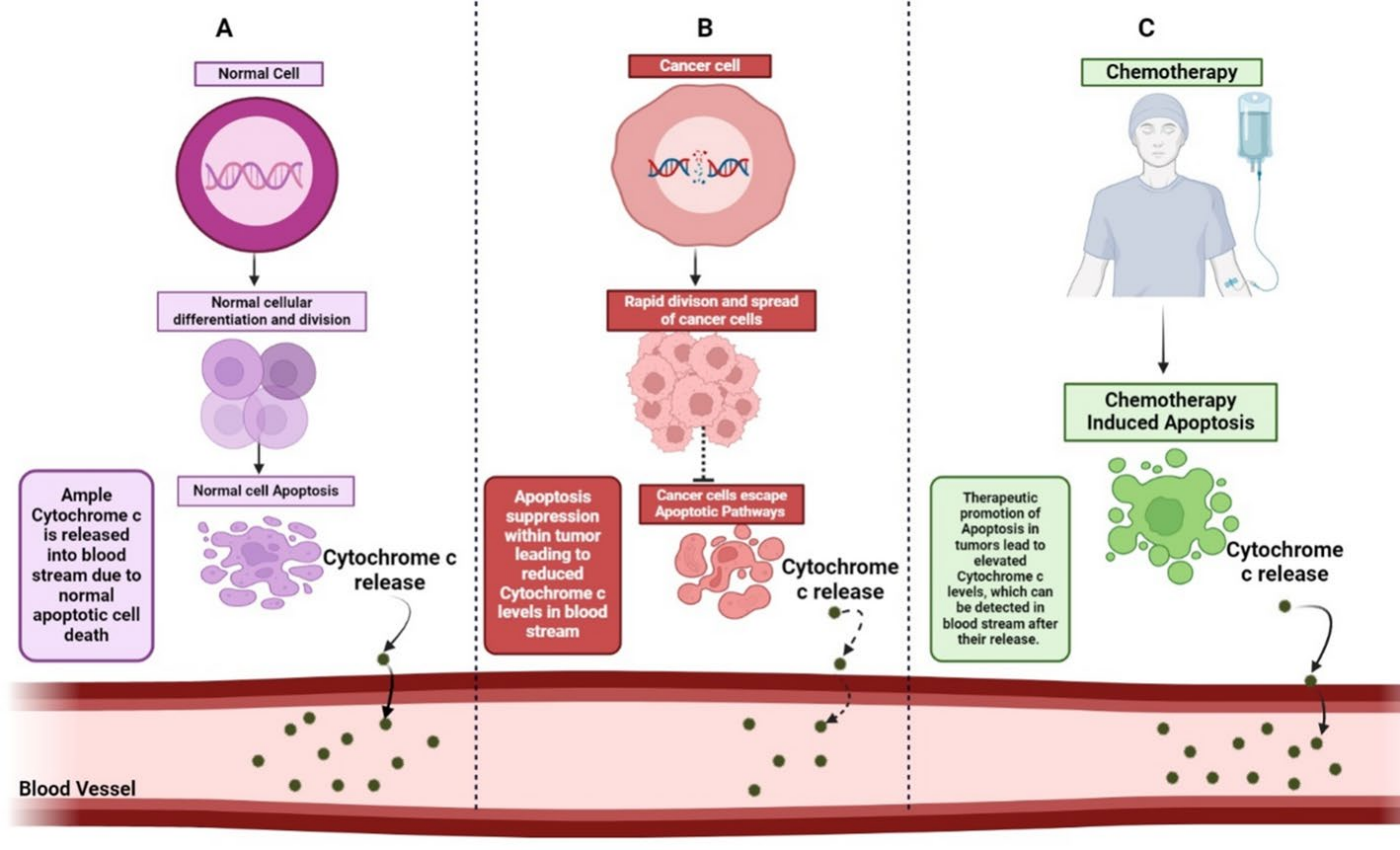
**A **Normal cell undergoing regular differentiation and Apoptosis, as result sufficient amount of Cytc is released out of mitochondria to blood stream. **B** Tumours suppress apoptosis, which correlates with a drop in Cytc levels in blood stream. **C **Cytoplasmic cyt C levels are raised as a result of therapeutically inducing apoptosis in tumours; these levels can subsequently enter the circulation. A good prognosis is linked to the finding of elevated Cytc levels in the serum

The amount of Cytc released can impact subsequent events. Research has shown that a living neural cell in rats can withstand damage to approximately 15% of its mitochondria through laser micro-irradiation. The Cytc released from these mitochondria wasn’t enough to trigger caspase-9 activation [99]. This indicates that there exists a critical concentration of Cytc, below which caspase-9 remains inactive. This threshold may vary significantly depending on the cell type, possibly due to variations in the levels of regulators like inhibitors of apoptosis proteins (IAPs) that modulate caspase activities. Rana et al. [100] have reported that the levels of Cytc expression are diminished in glioma tissues compared to those in normal tissues. What adds to the fascination is the observation that the expression level diminishes as glioma grades elevate. Consequently, this revelation indicates that Cytc could potentially serve as a focus for prognostic biomarkers in glioma.Additionally, it was seen that in a mouse model of clear cell renal cell carcinoma the overexpression of Cytc hindered the formation of tumors [101]. Conversely, the suppression of Cytc in a clear cell renal cell carcinoma cell line enhanced its growth, most likely by preventing programmed cell death. The overexpression of Cytc in these cells increased apoptotic rates [101]. In addition, numerous commonly used chemotherapeutic medications trigger apoptosis by facilitating the release of Cytc into the cytoplasm [102]. Thus, the rise in cytoplasmic Cytc levels is directly related to the ability to fight the disease.The exploration of Cytc expression levels across diverse malignancies remains relatively uncharted territory, presenting promising avenues for further investigation. This research endeavour could potentially establish Cytc as a valuable and dependable prognostic marker in cancer biology, marking a significant step forward in the field. An illustration of how the levels of Cyt c in blood stream may act as a potential marker for detection of carcinoma is depicted in Fig. 5.

### Exploring the future of cytochrome c targeting in cancer: prognostic and therapeutic innovations

Cytoplasmic Cytc can escape from the cell. It has been reported that, in samples of breast carcinoma, Cytc was consistently released from epithelial cells and entered the lumen of the malignant duct. Cytc is capable of evading cells, allowing it to enter the circulation [103]. Consequently, measuring its concentration in the serum can indicate its amount within the cytoplasm.Cancer has been found to cause a reduction in the amounts of Cytc in the blood. In patients diagnosed with non-small cell lung carcinoma, the levels of Cyt c in serum were almost three times lower compared to those of healthy individuals [104]. The levels of Cytc were reduced in the serum of patients with clear cell renal cell carcinoma [101]. These data suggest that the lack of apoptosis induction in cancer may be caused by insufficient levels of Cytc in the cytoplasm. However, serum Cytc levels can vary among different cancer patients. Despite the lack of a noticeable connection between the levels of Cytc in the blood and the stage of cancer, individuals with clear cell renal cell carcinoma who had greater levels of Cytc saw improved rates of survival compared to those with lower levels of the protein [101]. Elevated serum concentrations of Cytc in patients with different forms of cancer were found to be associated with a higher likelihood of patient survival. While elevated levels of serum Cytc can suggest increasing neoplasm content, their rise following cancer therapy has been associated with a more positive prognosis [40].Elevated blood levels of Cytc can also indicate heightened levels of apoptosis produced by chemotherapy [40]. In patients with non-small cell lung carcinoma [104], the level of serum Cytc raised a minimum of 13 times following the initial cycle of treatment. Chemotherapy has the potential to increase the levels of serum Cytc in individuals with malignancies of the blood, such as acute myeloid leukemia and non-Hodgkin lymphoma [37]. Patients with non-small cell lung carcinoma who had elevated amounts of Cytcbefore chemotherapy saw a greater spike in Cytc levels following the treatment [104]. These data suggest that chemotherapy led to elevated levels of serum Cytc in the patients and enhanced levels of tumor PCD.Chemotherapeutic medicines cause the outer membrane of mitochondria to become permeable, allowing the Cytc release into the cytoplasm and even into the extracellular media. This resulted in an elevated level of Cytc in the bloodstream. It has been suggested that in adult T-cell leukemia patients, the levels of serum Cytc are more responsive [37] The prompt reaction of Cytc levels to chemotherapy that induces PCD renders this protein an ideal indicator for evaluating the effectiveness of cancer treatments [105].Studies have shown that merging commonly utilized anti-cancer medications (vinblastine, vincristine, doxorubicin, paclitaxel, oxaliplatin, etc.) with hybrid nanoparticles adorned with Cytc for the treatment of liver carcinoma significantly enhances apoptosis in cell lines, ultimately resulting in cellular demise. Consequently, this combined approach holds promise for prospective therapeutic protocols [106].

## Capturing the essence: a recap of impact of cytochrome on apoptosis, cellular metabolism in line with cancer development

Extensive research on Cytc has yielded significant insights into both the respiration of mitochondria [107] and Apoptosis [33, 34] When exposed to pro-apoptotic signals, the mitochondrial outer membrane becomes permeable, allowing cytochrome c (Cytc) to be released into the cytosol, where it binds to Apaf-1 [108], which initiates a series of biochemical reactions activating the caspase cascade, a group of enzymes that drive apoptosis by dismantling cellular components [108, 109].Inhibition of apoptosis is a key feature of cancer that enables tumor cells to survive and proliferate despite cellular stress [110]. Furthermore, the initiation of cancer cell apoptosis holds remarkable therapeutic promise [111]. MOMP is regarded as an irreversible event that initiates the intrinsic pathway of PCD [105]. This leads to the release of cytochrome c (Cytc) from the intermembrane space into the cytoplasm. Lower Cytc levels observed in cancer tissues indicate apoptosis suppression. Alterations in cancer-related metabolic pathways are dynamic, adaptable, and often depend on the tumor type and its microenvironment, resulting in metabolic flexibility. Understanding these complex changes in metabolic flux can help guide the development of novel therapeutic strategies [74]. Studying Cytc’s role in apoptosis is important both biologically and clinically, as its cytoplasmic presence serves as a useful biomarker [112]. In cases of cellular damage, Cytc released into the extracellular space can act like other danger-associated molecular pattern molecules (DAMPs), performing similar functions when present in unintended locations [113]. Therefore, the detection of Cytc in extracellular spaces serves as a valuable indicator of severe mitochondrial damage leading to cell death.

Metabolic reprogramming in cancer, particularly the Warburg effect, promotes aggressive tumor progression by shifting energy metabolism toward aerobic glycolysis, enabling rapid ATP production and lactate accumulation. This metabolic shift not only supports cancer cell proliferation but also confers resistance to apoptosis, enhancing tumor survival. Dysregulated glucose metabolism, together with mutations in mitochondrial-associated genes, further drives tumorigenesis and sustains malignancy, while oxidative stress and redox imbalances contribute to continued tumor growth. Despite the variability in bioenergetic adaptations across cancer types, targeting central metabolic pathways such as glycolysis and the pentose phosphate pathway represents a promising therapeutic approach. The intricate interplay between metabolism and apoptosis underscores the complexity of cancer progression. Understanding these metabolic alterations provides critical insights for therapeutic intervention, with Cytc emerging as a potential target. Restoring Cytc-mediated apoptotic function may re-sensitize cancer cells to programmed cell death, inhibiting tumor growth and enhancing treatment efficacy. Thus, exploring the role of Cytc in cancer metabolism and apoptosis offers a promising strategy for developing targeted therapies and improving patient outcomes.

## Conclusion

Arguably, the act of cell demise stands as one of the most crucial decisions a cell can make, and consequently, the release of Cytc from the confines of mitochondria is controlled by multiple aspects of regulation. Significant efforts have been devoted to unveiling the crucial function of Cytc in the intrinsic pathway of cell deathand metabolic shift, the processes governing Cytc discharge from mitochondria, and the control mechanisms influencing this liberation and its consequences. However, there remains ample room for further understanding.Moreover, there is limited understanding regarding the degree of Cytc liberation, if any, in cells that evade death. The role of mitochondrial dynamics, bioenergetics, and other aspects of mitochondrial function in mediating MOMP and Cytc emission remains contentious, as do the impacts of Cytc alterations on apoptotic processes.

## Data Availability

No datasets were generated or analysed during the current study.

## Abbreviations

Cytc: Cytochrome c
COX: Cytochrome c oxidase
ETC: Electron transport chain
ROS: Reactive oxygen species
Apaf-1: Apoptosis protease-activating factor-1
DISC: Death-inducing signalling complex
OXPHOS: Oxidative phosphorylation
CL: Cardiolipin
MOMP: Mitochondrial Outer Membrane Permeabilization
PCD: Programmed Cell Death
OMM: Outer Mitochondrial Membrane
IMM: Inner Mitochondrial Membrane
